# Cytisine for smoking cessation in hospitalised smokers with cardiovascular diseases: an observational study

**DOI:** 10.1007/s11739-025-03888-5

**Published:** 2025-02-12

**Authors:** Erika Tedesco, Sofia Ceccato, Alessandro Torazzi, Laura Santin, Lorenzo Losso, Andrea Bottardi, Rebecca Casari, Silvia Melchiori, Erica Secchettin, Valeria Ferrero, Elena Arzenton, Paola Marini, Fabio Lugoboni, Cristiano Chiamulera

**Affiliations:** 1 Department of Diagnostic and Public Health, Pharmacology Section, University of Verona, Verona, Italy; 2 Department of Pharmacy, Verona University Hospital, Verona, Italy; 3 Department of Addiction Medicine, Verona University Hospital, Verona, Italy; 4 Department of Surgery, Dentistry, Paediatrics and Gynecology, University of Verona, Verona, Italy; 5 Department of Cardiology, Verona University Hospital, Verona, Italy

**Keywords:** Cytisine, Smoking cessation, Hospital, Cardiovascular disease, Addiction, Safety

## Abstract

Cigarette smoke is a significant risk factor for cardiovascular diseases (CVD). Among pharmacotherapy for smoking cessation, the plant alkaloid cytisine (CYT) -a nicotinic receptors partial agonist- has been shown to have a safe profile, with a minimal risk for drug interactions. Since previous studies have excluded CVD patients, there are few existing data examining CYT safety in this critical population. An observational prospective study was conducted in the Verona University Hospital (AOUI), Italy, to assess the safety and efficacy of CYT for smoking cessation. Thirty-six hospitalised participants from the Cardiology Department received oral CYT 1.5 mg for 25 days, according to the West Dosing Schedule (6 capsules for the first 3 days, gradually decreased up to 2 capsules on the last 6 days), in combination with supportive care. The primary endpoint was CYT safety, with 11 mild-moderate Adverse Drug Reactions (ADRs) reported by 9 (25%) participants. Initial insomnia (11%), nausea (6%), sleep disorders (6%), headache (3%), gastritis (3%), and diarrhoea (3%) were the most frequent symptoms. No serious or unexpected ADRs were identified, with no increase in cardiovascular events. Efficacy was assessed as self-reported 7-day point prevalence abstinence (PPA) at 3, 6 and 12 months post-quit. At the first follow-up, abstinence was also biochemically verified by exhaled carbon monoxide (CO) measurement, which was confirmed for 36% of participants. Considering lost to follow-up as relapsed patients, the PPA was 50%, 47% and 36% at 1st, 2nd and 3rd follow-up, respectively. These results may suggest that CYT has a well-established safety profile in hospitalised CVD patients, but further investigation is needed.

**Supplementary Information:**

The online version contains supplementary material available at 10.1007/s11739-025-03888-5.

## Introduction

Cardiovascular diseases (CVD) represent a worldwide public health concern, contributing to 30% of all deaths [1]. Various risk factors could raise the incidence of CVD, including poor dietary habits, physical inactivity, environmental pollution and tobacco consumption [1]. Tobacco smoke is recognized as a significant risk for atrial fibrillation, peripheral vascular disease, strokes, and sudden cardiac death [2, 3] and the risk increases with more extended usage periods and with the number of cigarettes daily smoked [4, 5]. The intake of several chemicals by tobacco smoking, such as nicotine, carbon monoxide (CO) and other oxidant compounds are often related to CVD development [4, 6]. In addition, through its binding to nicotinic acetylcholine receptors (nAChRs), nicotine triggers dopamine release, a neurochemical effect that plays a major role in tobacco addiction [7, 8]. Nicotine withdrawal symptoms negatively impact abstinent smokers daily functioning, increasing the risk of relapse [9]. The majority of smokers struggle to remain abstinent, with 95% of self-quitting attempts resulting in failure within six months [10] and 40% of patients with CVD resume smoking within one year [11], frequently even while they are still in the hospital [12, 13]. Since many smoke-related chronic illnesses, such as CVD, require hospitalisations, hospitals could represent an ideal setting to begin an intervention for smoking cessation [14], to safeguard cardiovascular health and reduce the incidence of other smoke-related diseases [4, 7, 9].

Pharmacotherapy has proven to be an effective approach for smoking cessation [15, 16]. Nicotine Replacement Therapy (NRT) and non-NRT represent the two main categories of pharmacological interventions [16]. Varenicline and cytisine (CYT) have a high-affinity for the α4β2 (nAChRs) subtype and act as partial agonists, thus maintaining moderate levels of dopamine and lowering smoking satisfaction [17–20].

CYT is a plant-based quinolizidine alkaloid found in seeds of members of the *Leguminosae* family (especially *Laburnum anagyroides*) [21, 22]. Bulgaria and Poland use CYT as a smoking cessation intervention since the 1960s [23–25] and many studies have demonstrated the safety and efficacy of CYT treatment [26–28] and superior efficacy compared to NRT [22]. Throughout more than forty years of CYT utilization as a smoking cessation aid, no serious adverse events (AEs) have been documented at therapeutic levels [23, 29]. CYT has a short plasma half-life (4.8 h) and after oral administration is eliminated unchanged through kidneys with no hepatic metabolism [23, 30, 31], with a limited risk of drug-drug interactions. Furthermore, the reduced affinity of CYT for 5-HT3 receptors leads to a lower incidence of nausea compared to varenicline, making it particularly advantageous [32] for hospitalised patients, frequently susceptible to gastrointestinal symptoms. Thus, CYT may represent a promising option for hospitalised smokers with concomitant pathologies and/or on polypharmacotherapy.

Some previous studies excluded participants with contraindicated medical conditions according to the CYT label (arterial hypertension or advanced arteriosclerosis) [12], with systolic/diastolic blood pressure above 150/100 mmHg, with self-reported cardiovascular event in the 2 weeks before study enrollment [22], or with clinically significant medical comorbidities [29]. A priori exclusion of these participants could hinder the potential inference of efficacy and safety findings to patient populations with a wide range of comorbidities. However, since a recent study showed that CYT is safe in patients with coronary artery disease [33], we hypothesized that CYT could be a safe smoking cessation intervention in hospitalised patients, including those with CVD. We performed a subgroup analysis of an observational study conducted on smokers admitted to the local Verona University Hospital (AOUI), Italy, which also involved the Cardiology Department. The aims were, primarily to investigate the safety profile of CYT treatment for smoking cessation and, secondly, to assess the efficacy and patients’ compliance with the treatment. The findings of this study will improve knowledge concerning CYT management in a hospital setting, with a focus on patients with CVD, and promote its use in an integrated intervention that combines smoking cessation with relapse prevention.

## Methods

### Study design

Hospitalised smokers with CVD are particularly critical and frail since comorbidities and polypharmacotherapy are common. Given these aspects, we decided to conduct a subgroup analysis of a specific patient population enrolled in a single-centre observational prospective study (CITOSP trial) conducted in the AOUI from November 2021 to August 2023 [34]. The population considered in this analysis included smokers admitted to the Cardiology Department.

The study received Local Ethics Committee approval on September 23rd 2021 (reference protocol number 55360-3439CESC) and was registered in the Observational Study Registry. Good Clinical Practice guidelines (ICH GCP) and the Declaration of Helsinki were followed in the way of conducting and monitoring the trial and all participants provided written informed consent.

### Setting and Participants

Patients admitted to the AOUI Cardiology Department for CVD were screened for active smoking. Eligible participants were adult smokers (18 years or older), willing to assume CYT for smoking cessation, available for a 12-month follow-up and able to provide written informed consent. The exclusion criteria were: reported hypersensitivity to CYT, pheochromocytoma, malignant hypertension, persistent unstable angina despite best therapy, pregnancy or breastfeeding.

At baseline, demographic information, clinical anamnesis, smoking history and nicotine dependence level were collected. AEs, suspected Adverse Drug Reactions (ADRs), smoking abstinence and CYT compliance were assessed at each control visit, 7 and 25 days after the Quit Day (QD), and at each follow-up, 3, 6 and 12 months after the QD. Patients who failed to attend visits or follow-up appointments were considered lost to follow-up.

### Endpoint and outcome measures

The primary endpoint was to evaluate the safety of CYT treatment. Secondary endpoints included patient compliance with CYT treatment and its efficacy.

According to the EMA *Guideline on Good Pharmacovigilance Practices* (GVP), AEs are defined as “any harmful clinical event that occurs in a patient or in a person involved in a clinical trial that has been given a medicinal/device product and does not necessarily have a causal relationship with this treatment”, while ADRs stand for “all untoward and unintended responses to an investigational medicinal product related to any dose administered” [35].

AEs reported by participants were collected to assess CYT safety profile. At each visit or follow-up, a Clinical Monitor (CM) recorded all AEs in the electronic Case Report Form (eCRF) RedCap® [36, 37]. If an AE occurred, the physician evaluated the event-treatment relationship to recognise a suspected ADR. In case of a suspected ADR, the physician or the CM filled out the online Italian Medicines Agency (AIFA) spontaneous reporting form. The report included a summary of the textual description of the suspected ADRs, coded using the Medical Dictionary for Regulatory Activities (MedDRA) terminology.

The efficacy of CYT treatment was evaluated as self-reported 7-day point prevalence abstinence (PPA, no cigarettes, not even a puff in the previous 7 days) at 3-, 6- and 12-months post-QD. Smoking status was also recorded after 7 and 25 days after QD. Patient self-reporting and a standardised exhaled carbon monoxide (CO) measurement using a Bedfont Smokerlyzer® (Bedfont Scientific Ltd, Kent, UK) were used to confirm abstinence during the first two visits and at the first follow-up (3 months). A level of CO < 6 ppm confirmed abstinence. In the case of missing CO data, self-reported abstinence was taken into consideration. According to West et al. (2011) [12], measuring CO levels is a reliable indication of smoking cessation over a 48–72-h period. At the second and third phone call follow-up (6 and 12 months), efficacy was assessed by reported 7-day PPA[12, 38].

A relapse occurred when patients smoked just one puff in the previous 7 days before the 3-, 6- and 12-month follow-up. Participants missing follow-up visits (lost to follow-up) were considered relapsed.

Participant completion of a daily dosage diary for drug accountability or self-reported adherence was used to assess study CYT compliance.

### Recruitment and cytisine treatment

During the screening, patient's eligibility was assessed, according to inclusion and exclusion criteria. The CM described the study in clear and plain language, and, if patients were interested in participating, written informed consent was sought before the completion of the baseline interview.

Baseline data included general demographic information such as age, sex, highest level of education achieved, and current employment status. A comprehensive medical history was obtained, including information on concomitant disorders, current and past anamnesis, and concurrent medications. Details about participant smoking behaviours, such as age of smoking initiation, number of daily smoked cigarettes, and previous quitting attempts, were collected. Furthermore, the Fagerström Test for Cigarette Dependence Questionnaire (FTND) [39] was used to measure nicotine dependence.

After baseline data collection, participants received CYT supplied by Hospital Pharmacy (HP) as 1.5 mg galenic capsules, along with comprehensive counselling. In case of discharge, CYT capsules were supplied for the whole treatment and patients continued their therapy at home. In accordance with the West dosing schedule [12] CYT 1.5 mg capsules were administered orally in a dose of 1 capsule every 2 h (6 capsules daily) for 3 days; from the 4th to 12th day—1 capsule every 2.5 h (5 capsules daily); from the 13th to 16th day—1 capsule every 3 h (4 capsules daily); from 17 to 20th—1 capsule every 4 h (3 capsules daily), from the 21st to 25th day—1 capsule every 6 h (2 capsules daily) [12, 40]. According to published reports and protocols, the 25-day West dosing schedule is the most used up to today’s date.

The pharmacological intervention with CYT was integrated into the Treatment As Usual (TAU) provided by the Addiction Medicine Department (AMD). In accordance with clinical practice, after the clinical stabilisation of smokers, ward staff requested the AMD intervention for anamnesis and smoking history assessment. Once patient’s clinical condition and needs have been evaluated, an individualised programme that combines psychological support and/or pharmacological intervention (such as nicotine patches or CYT capsules) was proposed. The decision for the most appropriate intervention was based on several factors, including a comprehensive risk–benefit assessment. The prescription of the pharmacological intervention for smoking cessation was followed by proper counselling. Follow-ups after discharge were scheduled to provide continuous support, including behavioural strategies to enhance motivation and treatment adherence, with praise in case of successful abstinence. Additionally, practical advice was also given to overcome craving symptoms. These interventions occurred during both in-person visits and telephonic follow-ups.

### Visits and follow-up

The study timeline consisted of one baseline visit before QD (for screening and enrolment), two in-person control visits (on day 7 and day 25 of CYT treatment, Visit 1 and Visit 2, respectively) and 3 follow-up visits 3, 6, and 12 months following the QD (Visit 3, Visit 4 and Visit 5). The first follow-up took place in person, while the last two follow-ups (at 6 and 12 months after QD) were done by phone call. If participants could not attend in person for the first follow-up, the visit was conducted by phone call, recording all study-defined measures, except for CO measurement.

### Statistical analysis

Considering a frequency of 30% of suspected ADRs and by using the calculator proposed by Viechtbauer et al. for pilot studies [41] with a confidence interval of 0.80 and an alpha of 0.05 and adding up to 17% drop out and lost at follow-up (derived from local data and consistent with the literature [22, 33]), the required sample size was of 36 patients to be recruited. Participant enrollment was conducted progressively, according to the Cardiology Department feasibility criteria. From November 2021 to August 2023, the number of participants admitted to the AOUI Cardiology Department and enrolled in the CITOSP study was 36, as suggested from a priori estimation.

Continuous variables are reported as means and standard deviations, while categorical variables are expressed as percentages and proportions.

To evaluate CYT treatment safety, the frequency of AEs and ADRs up to day 7, day 25 and up to a year after QD was recorded. All participants who assumed at least one capsule of CYT were considered for the analysis.

The proportion of participants who assumed CYT and maintained abstinence from smoke in the previous 7 days before each follow-up was considered to assess treatment efficacy. Additionally, the median number of cigarettes smoked daily in the case of relapsed patients was evaluated at each study timepoint.

## Results

### Patients’ characteristics

**Fig. 1:**
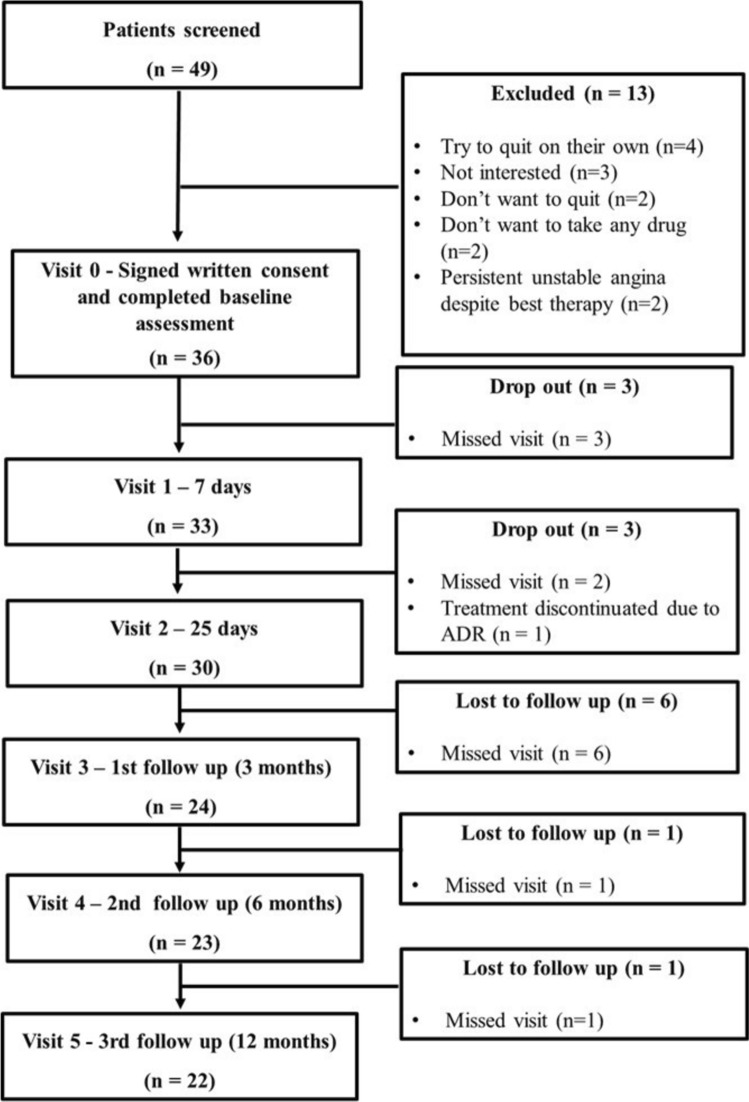
Flow diagram of sample recruitment in the Cardiology Department. Screening, enrolments, exclusion from the trial and lost to follow-up. *ADR* Adverse Drug Reaction

In the Cardiology Department, a total of 49 patients were screened, of which 36 (73.5%) submitted written informed consent. Thirteen (26.5%) of 49 patients declined study participation due to different reasons. Of these, 4 (30.8%) wanted to quit on their own, 3 (23.1%) were not interested, 2 (15.4%) did not want to quit, 2 (15.4%) did not want to take any drug for smoking cessation, 2 (15.4%) showed persistent unstable angina. The flow diagram of study sample recruitment is reported in Fig. 1.

During the study, 14 (38.9%) of 36 patients discontinued visits or follow-up at different time points and were considered drop out or lost to follow-up, while 22 (61.1%) of 36 participants completed the study. In particular, 6 (16.7%) patients discontinued during CYT treatment (3 at Visit 1, and 3 at Visit 2), while 8 (22.2%) patients discontinued during follow-up (6 at 1st follow-up, 1 at 2nd follow-up and 1 at 3rd follow-up).

Table 1 describes the participants demographic characteristics of patients at baseline visit. The median age of the patients at enrolment was 56.75 ± 9.63 (Mean ± SD) years, and 83.3% were male. Patients started smoking on average at age 16.03 ± 3.48 (Mean ± SD), and in the last year they smoked an average of 21.42 ± 12.23 (Mean ± SD) cigarettes per day. Twenty-eight (77.8%) patients had previously tried to quit. The mean score of the FTND Questionnaire was 5.31 ± 2.56 (Mean ± SD).

**Table 1:** Baseline demographic characteristics of the study participants

	All
	*N* = 36
**Demographic**	
Age, years *Mean* ± *SD*	56.75 ± 9.63
Sex	
Males *n* (%)	30 (83.3)
Females *n* (%)	6 (16.7)
**Smoking history**	
Start smoking age *Mean* ± *SD*	16.03 ± 3.48
Cigarettes per day *Mean* ± *SD*	21.42 ± 12.23
Previous attempts to quit	
YES *n* (%)	28 (77.8)
NO *n* (%)	8 (22.2)
FTND score *Mean* ± *SD*	5.31 ± 2.56
**Clinical comorbidities (SOC)*** n patients (%)*	
Cardiac disorders	29 (80.6)
Vascular disorders	20 (55.6)
Metabolism and nutrition disorders	15 (41.7)
Gastrointestinal disorders	6 (16.7)
Surgical and medical procedures	4 (11.1)
Psychiatric disorders	3 (8.3)
Neoplasms benign, malignant and unspecified (incl cysts and polyps)	3 (8.3)
Nervous system disorders	3 (8.3)
Respiratory, thoracic and mediastinal disorders	3 (8.3)
Reproductive system and breast disorders	2 (5.6)
Endocrine disorders	2 (5.6)
Blood and lymphatic system disorders	1 (2.8)
Eye disorders	1 (2.8)
Infections and infestations	1 (2.8)
Injury, poisoning and procedural complications	1 (2.8)
Skin and subcutaneous tissue disorders	1 (2.8)

*FTND* Fagerström Test for Cigarette Dependence Questionnaire; *SD* Standard Deviation; *SOC* System Organ Class classification

Clinical comorbidities were summarised and expressed according to the System Organ Class (SOC) classification. “Cardiac disorders” were the most common comorbidities and included 29 (80.6%) patients, followed by “Vascular disorders” (55.6%) and “Metabolism and nutrition disorders” (41.7%). Many patients reported more than one comorbidity, which could be included in the same or in a different SOC. For the three most frequent SOCs, a detailed description considering the Preferred Term (PT) classification was reported in Table 2. For “Cardiac disorders” the most reported PT was “Acute myocardial infarction”, for “Vascular disorders” was “Hypertension”, while “Hypercholesterolaemia” was the most frequent PT for the SOC “Metabolism and nutrition disorders”.

**Table 2:** Preferred Terms included in the most three System Organ Classes reported by study participants

	All
	*N* = 36
**Cardiac disorders PTs**, *n patients (%)*	
Acute myocardial infarction	18 (50.0)
Myocardial ischaemia	4 (11.1)
Cardiac failure	3 (8.3)
Angina pectoris	2 (5.6)
Atrial fibrillation	2 (5.6)
Cardiomyopathy	2 (5.6)
Coronary artery occlusion	1 (2.8)
Acute coronary syndrome	1 (2.8)
Cardiac arrest	1 (2.8)
Cardiac disorder	1 (2.8)
Cardiac failure chronic	1 (2.8)
Hypertensive heart disease	1 (2.8)
Ischaemic cardiomyopathy	1 (2.8)
Mitral valve prolapse	1 (2.8)
Myocardial infarction	1 (2.8)
Tachycardia paroxysmal	1 (2.8)
**Vascular disorders PTs**, *n patients (%)*	
Hypertension	15 (41.7)
Arteriosclerosis	2 (5.6)
Aortic aneurysm	1 (2.8)
Arterial stenosis	1 (2.8)
Deep vein thrombosis	1 (2.8)
**Metabolism and nutrition disorders PTs**, *n patients (%)*	
Hypercholesterolaemia	6 (16.7)
Hyperlipidaemia	5 (13.9)
Type 2 diabetes mellitus	5 (13.9)
Dyslipidaemia	2 (5.6)
Obesity	2 (5.6)
Diabetes mellitus	2 (5.6)
Hyperuricaemia	1 (2.8)
Overweight	1 (2.8)

*SOC* System Organ Class classification; *PT* Preferred term classification

Concomitant therapies were recorded using the Anatomical Therapeutic Chemical (ATC; as shown as classification name and alpha-numeric code) classification system. For all patients, a total of 234 drugs were recorded during baseline visit. Patients assumed an average of 6.50 ± 3.04 (Mean ± SD) different medications due to their comorbidities and 28 (77.8%) of 36 patients assumed 5 or more medications. Most frequent medications were “Antithrombotic Agents, B01” (21.4%), “Lipid Modifying Agents, C10” (13.7%) and “Drugs For Acid Related Disorders, A02” (11.5%). The detailed report of drug frequency is available in Table 1S of Supplementary Materials.

### Safety

During the study period, 19 (52.8%) of 36 patients reported at least one AE, for a total of 34 AEs. Among these, 23 (67.7%) were identified by the physician as "not CYT related" and not considered as suspected ADRs. Five of these "not CYT related" AEs were serious and required hospitalisation. One serious AE occurred after 3 months from QD (neurosurgical intervention for brain aneurysm diagnosed during a follow-up CT scan), and 2 serious AEs occurred after 6 months from the QD (syncope with a gastroenterological diagnosis; unstable angina in a patient with chronic ischemic cardiomyopathy) so there was no temporal correlation between CYT treatment and these events. Two serious AEs occurred during CYT treatment and were considered related to patients’ pre-existing clinical conditions: one patient was admitted for pulmonary oedema, attributed to the decline of the ventricular function due to a recent myocardial infarction, while the other patient experienced glycaemic imbalance due to diabetes.

Eleven (32.4%) of 34 AEs reported by 9 (25.0%) of 36 participants were considered potentially related to CYT and were classified as suspected ADRs. Overall, the ADRs collected (Table 3) during the study were: initial insomnia, nausea, sleep disorder, headache, gastritis and diarrhoea. No unexpected ADRs were identified and no related cardiovascular reactions reported. Nine (81.8%) of 11 suspected ADRs occurred in the first seven days of therapy, when CYT dosage was higher, while the remaining 2 (18.2%) occurred from day 7 to day 25 after QD. One (2.8%) participant discontinued study treatment earlier due to ADR and for 2 (5.6%) patients CYT dosage reduction was decided due to treatment-related symptoms. At 3–6–12 months no suspected ADRs were recorded.

**Table 3:** Types of suspected ADRs (Preferred Term classification level) and their frequency in participants during CYT treatment and the entire period of follow-up

	Participants
	*N* = 36
**ADR**	*n* records (%)
Initial insomnia	4 (11.1)
Nausea	2 (5.6)
Sleep disorder	2 (5.6)
Headache	1 (2.8)
Gastritis	1 (2.8)
Diarrhoea	1 (2.8)

*ADR* Adverse Drug Reaction

### Efficacy

At the first follow-up, 18 (75.0%) of 24 patients reported abstinence from smoking, while 6 (25.0%) of 24 referred to cigarette smoking in the previous 7 days. Fifteen (62.5%) of 24 patients attended follow-up visits in person, of which 13 (86.7%) confirmed biochemically their abstinence status and 2 (13.3%) confirmed their smoking status. The remaining 9 (37.5%) of 24 patients carried out the follow-up visit by telephone.

At the second follow-up, 17 (73.9%) of 23 patients reported abstinence from smoking, while 6 (26.1%) relapsed in the 7 days preceding the visit. One year after QD, 13 (59.1%) of 22 patients reported abstinence from smoking, while 9 (40.9%) smoked in the previous 7 days.

**Fig. 2:**
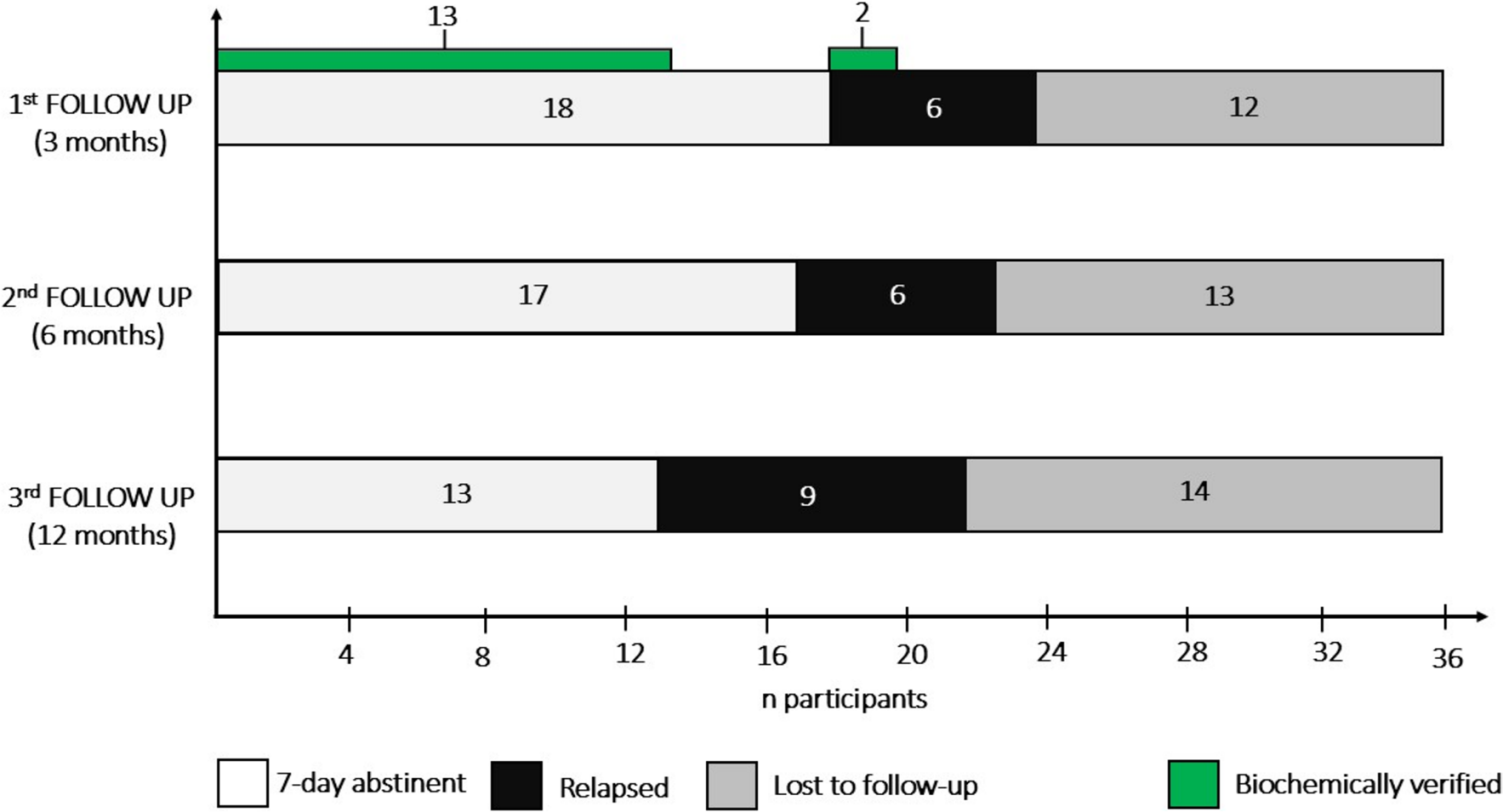
Horizontal bars represent CYT efficacy data expressed as self-reported Point Prevalence Abstinence, biochemically confirmed at the first follow-up through measurement of exhaled carbon-monoxide

Considering lost to follow-up as relapsed, the PPA was equal to 50.0% (18/36), 47.2% (17/36) and 36.1% (13/36) at the 1st, 2nd and 3rd follow-up, respectively (Table 4, Fig. 2).

**Table 4:** Data of CYT treatment efficacy expressed as Point Prevalence Abstinence, carbon-monoxide objective measurement and mean number of cigarettes smoked per day. For the last two follow-up (6 and 12 months) carbon-monoxide measurement was not carried out because visits were telephonic

	N = 36	1st follow-up	2nd follow-up	3rd follow-up
Visits recorded	*n* (%)	24 (66.7)	23 (63.9)	22 (61.1)
Lost to follow up (LFU)ǂ	*n* (%)	12 (33.3)	13 (36.1)	14 (38.9)
PPA*	*n* (%)	18 (75.0)	17 (73.9)	13 (59.1)
PPA (with LFU)	%	50.0	47.2	36.1
CO° measurement	*n* (%)	15 (62.5)	/	/
CO ≤ 6 ppm	*n* (%)	13 (86.7)	/	/
6 ppm < CO ≤ 8 ppm	*n* (%)	0 (0.0)	/	/
CO ≥ 9 ppm	*n* (%)	2 (13.3)	/	/
CPD**	*Mean* ± *SD*	12.3 ± 10.6	12.2 ± 10.4	9.1 ± 10.4
CPD reduction rate (vs CPD baseline)	*%*	42.4	43.2	57.8

ǂ Lost to follow up

^*^ Point Prevalence Abstinence

° Carbon Monoxide

^**^Cigarettes per day

At each follow-up, the number of cigarettes smoked per day (CPD) was recorded to monitor changes over time in case of relapse. The mean number of CPD were 12.3, 12.2 and 9.1, with reduction rates of 42.4%, 43.2% and 57.8% at 3, 6 and 12 months after QD, respectively (Table 4). Notably, only one participant exhibited an increase in CPD from 25 to 30 over the observation period.  In all other cases, the reduction in CPD consistently exceeded 50%.

### Compliance

At the end of the treatment, compliance with the CYT Dosing Schedule was assessed and 10 (27.8%) patients committed at least one dosing schedule violation. Three (8.3%) patients reduced or stopped CYT’s administration due to ADR symptoms: in particular, drug reduction occurred for 2 (5.6%) and drug discontinuation for 1 (2.8%). Seven (19.4%) patients missed a variable number of CYT capsules (> 2 cps).

## Discussion

Hospitalisation for a cardiac event represents a crucial and teachable event for smokers, who face the risks and consequences of smoke and could be more motivated to quit. In these circumstances, providing safe and effective smoking cessation intervention is essential [42].

Substantial concerns about the cardiovascular safety of smoking cessation interventions have been raised previously [43]. The double-blind Evaluating Adverse Events in a Global Smoking Cessation Study (EAGLES) was designed to assess the cardiovascular safety of smoking cessation therapies, comparing varenicline and bupropion with NRT patch and placebo, with no evidence that these pharmacotherapies increased the risk of serious cardiovascular adverse events [44]. These results were consistent with previous studies [45–48] and meta-analysis [49, 50].

About CYT, a partial agonist of the α4β2 nicotinic acetylcholine receptor (like varenicline), there is a lack of knowledge about its use in hospitalised smokers or patients with particular clinical conditions, such as CVD [51]. An exception is a recent study about CYT safety in patients with coronary artery disease [33], which reported no differences in the frequency of all AEs between treatment and control groups. Given that hospitalised active smokers with CVD are often frail and characterized by comorbidities and polypharmacotherapy, we deemed it necessary to investigate the CYT safety profile in this population.

Based on findings from our study, CYT was well tolerated, with only 2.8% of treatment discontinuation due to ADRs. The majority of suspected ADRs occurred in the first few days after the initiation of CYT treatment, during which the CYT daily dosage is higher compared to the following days. The most reported ADRs were related to sleep disorders and gastrointestinal symptoms, in accordance with previous literature on CYT usage [17, 24, 51–54] and with the summary of product characteristics [55]. It is noteworthy to acknowledge that some clinical manifestations, such as sleep disturbances, may be likely attributed to nicotine withdrawal rather than to CYT [23, 56]. Importantly, no serious suspected ADRs, such as cardiovascular events, directly attributable to CYT treatment were identified during our observation. The frequency of CYT-related side effects reported in the literature varies widely, ranging from a ratio of 0.36 [57] to 1.47 [52]. In our study, the frequency ratio was 0.31 for suspected ADRs and 0.94 when including all events, even those deemed unlikely to be related to CYT, aligning with the wide range of frequencies reported in the literature.

Our data showed self-reported abstinence rates of 50.0%, 47.2%, and 36.1% at 3, 6, and 12 months after the QD, respectively, even if assuming lost to follow-up as relapsed patients may underestimate the efficacy of the intervention and may overestimate the relapse rates. Excluding lost to follow-up, cessation rates were higher: 75.0%, 73.9%, and 59.1% at each follow-up, respectively, highlighting the significance of supportive care offered during follow-up visits. Our abstinence rates are similar to those reported in prior studies in which CYT efficacy ranged from 30% [22] to 52% [58]. It is however important to remark that CO measurements were only taken on days 7 and 25 after QD and at the first follow-up. There is therefore inconsistency between short-term CO-verified abstinence at the first follow-up visit and the longer-term self-reported abstinence data. We acknowledge this limitation as it impacts the ability to robustly correlate short- with long-term outcomes. While our study provides preliminary evidence supporting the safety and potential efficacy of CYT in a high-risk population, these limitations prevent to estimate its long-term impact.

Considering that 27.8% of patients violated the West dosing schedule, it is important to consider its feasibility, especially in the case of polypharmacotherapy. Recent studies assessed the efficacy and safety of different CYT dosing regimens. A placebo-controlled randomized clinical trial introduced a pharmacokinetically based CYT dosing regimen for 6 or 12 weeks with significant efficacy and safety in treating tobacco addiction [59]. Other recent observational studies assessed a 40-days CYT treatment (with an induction phase and a slower reduction schedule) with promising results in terms of efficacy rate and safety profile [60, 61]. As reported above, the Pozzi et al. study [60] by using the gradual dosing regimen (starting with two 1.5-mg tablets/first day, then daily scaling-up to six tablets/day on 8th to 14th day, and then weekly dose scaling-down until days 36th to 40th), showed a 16% rate of drop-out. We could hypothesize that gradual scaling up and then scaling down of CYT dosing might help to retain more individuals in treatment thanks to a more gradual ‘experience’ to the treatment. Treatment duration is therefore a still open issue about CYT treatment for smoking cessation. The lesson that could be drawn from previous clinical trials is that when CYT was compared to varenicline failed to significantly reach non-inferiority when given for 25 days but showed equal efficacy of varenicline after 84 days of treatment [62]. This is a finding that should be taken into consideration for further studies, as well future comparative investigations. In fact, comparative studies would be also needed in order to compare CYT to different NRT formulations (Walker et al. [22] showed a non-inferiority of CYT vs. NRT), bupropion slow release, and other emerging non-pharmacological intervention for smoking cessation. Considering all these aspects, it may be important to explore alternative CYT dosing schedules or formulations (e.g., prolonged-release formulations in order to reduce the need for multiple daily administrations) to enhance treatment adherence and optimize therapeutic benefits. Additional data should be collected not only by comparing pharmacological and non-pharmacological agents for smoking cessation, but also in different medical real-world context, as recommended by the Italian Guideline for Smoking Cessation [63]: randomized-controlled trials with different modes of dosage, galenic formulations, and in patients subgroups, not motivated to quit, difficult smoking subjects.

Our study presents several limitations. Firstly, this is a subgroup observational analysis, with a small sample size, without placebo or control group. This is a limitation for inference to the corresponding smoker population with CVD, and to smoker general population, that suggest a careful attention to generalized prescription of CYT. In fact, the participants in this study have been exposed to a multifactorial intervention that beside the pharmacological effects of CYT (whether on safety or efficacy) may have affected motivation and compliance. We are aware of this limitation that stress the need for further analysis in other hospitalised patients’ groups in order to identify similarity and differences.

Another limitation of the study is that the distinction between AEs and suspected ADRs was made by the study clinicians. This approach may have introduced a potential bias in the assessment of the events. To mitigate potential bias in future studies, the distinction between AEs and ADRs could be eliminated, reporting all events as potentially related to the treatment, or assigning the AEs evaluation task to an independent clinician, not directly involved in the study.

Additionally, although internal and literature data estimated a loss of follow-up percentage between 10 and 20%, we had a higher number of subjects who discontinued, limiting the generalizability of the results. Finally, the study was carried out during the COVID-19 pandemic, which had an impact on both the enrolment and dropout rates, due to restricted hospital access, reduced staff availability, resource allocation and logistical difficulties.

Despite these limitations, our data may contribute with preliminary insights into the safety profile of CYT among hospitalised smokers with CVD, suggesting a potentially positive trend in patients in whom the drug is still contraindicated. Although proof of its effectiveness and safety in the general population, CYT is still not widely recognized as a smoking cessation treatment, especially in Western Europe [51, 64]. Anyway, thanks to its potential as an affordable, safe, and helpful smoking cessation drug, it is now of great interest [23, 40], also in Italy [63, 65]. In addition, varenicline products are no longer available, since the Regulatory Authority Food and Drug Administration (FDA) decided for products recall due to N-nitroso-varenicline impurity exceeding the acceptable daily intake limit [66]. To date, there is no known risk for impurities of nitrosamine derivatives for CYT, which could be considered safe also from this point of view.

Unlike other trials, that impose extensive exclusion criteria aligned with manufacturer precautions, our study provides preliminary clinical data about the cardiovascular safety of CYT in individuals with CVD. It is necessary to emphasize that to evaluate long-term CYT safety and efficacy, especially in vulnerable populations, high-quality RCTs with long-term follow-up are required. In addition, there is a need for research exploring the safety profile of CYT among populations with other comorbidities, such as renal failure.

## Conclusions

Our study, in spite of the limitations described above, is, however, an additional evidence on the safety and efficacy properties of CYT, in agreement with the findings of past studies. Providing efficient pharmacotherapy in smokers with high but perhaps avoidable risk of cardiovascular health is extremely important. Our results were supportive of an acceptable safety profile of CYT as a treatment for tobacco addiction in CVD hospitalised smokers, also in case of co-morbidity and polypharmacotherapy, if further research or clinical experience will validate and support our results.

## Supplementary Information

Below is the link to the electronic supplementary material.

Supplementary file1 (DOCX 15 KB)
