# Correction: Cytisine for smoking cessation in hospitalised smokers with cardiovascular diseases: an observational study

**DOI:** 10.1007/s11739-025-04067-2

**Published:** 2025-08-18

**Authors:** Erika Tedesco, Sofia Ceccato, Alessandro Torazzi, Laura Santin, Lorenzo Losso, Andrea Bottardi, Rebecca Casari, Silvia Melchiori, Erica Secchettin, Valeria Ferrero, Elena Arzenton, Paola Marini, Fabio Lugoboni, Cristiano Chiamulera

**Affiliations:** 1 Department of Diagnostic and Public Health, Pharmacology Section, University of Verona, Verona, Italy; 2 Department of Pharmacy, Verona University Hospital, Verona, Italy; 3 Department of Addiction Medicine, Verona University Hospital, Verona, Italy; 4 Department of Surgery, Dentistry, Paediatrics and Gynecology, University of Verona, Verona, Italy; 5 Department of Cardiology, Verona University Hospital, Verona, Italy

**Correction: Internal and Emergency Medicine (2025) 20:817–828** 10.1007/s11739-025-03888-5

In this article all authors given and sur names were inter changed.

The correct names are given below:

Erika Tedesco

Sofia Ceccato

Alessandro Torazzi

Laura Santin

Lorenzo Losso

Andrea Bottardi

Rebecca Casari

Silvia Melchiori

Erica Secchettin

Valeria Ferrero

Elena Arzenton

Paola Marini

Fabio Lugoboni

Cristiano Chiamulera

The original article has been corrected.

