# Supplementary material for: Cytisine for smoking cessation in hospitalised smokers with cardiovascular diseases: an observational study
Source: Intern Emerg Med. 2025 Feb 12;20(3):817–28. doi: 10.1007/s11739-025-03888-5 (PMC12009246; doi:10.1007/s11739-025-03888-5)
Supplement: Supplementary file 1 — Supplementary file1 (DOCX 15 KB) [file 11739_2025_3888_MOESM1_ESM.docx]

|  | **All** |
| --- | --- |
|  |  |
|  | ***N* = 234** |
| **Second level ATC*** | *n* records (%) |
| Antithrombotic agents | 50 (21.4) |
| Lipid modifying agents | 32 (13.7) |
| Drugs for acid related disorders | 27 (11.5) |
| Agents acting on the renin–angiotensin system | 24 (10.3) |
| Beta blocking agents | 23 (9.8) |
| Diuretics | 18 (7.7) |
| Drugs used in diabetes | 13 (5.6) |
| Calcium channel blockers | 7 (3.0) |
| Psycholeptics | 7 (3.0) |
| Urologicals | 5 (2.1) |
| Cardiac therapy | 4 (1.7) |
| Immunosuppressants | 4 (1.7) |
| Antidiarrheals, intestinal anti-inflammatory/anti-infective agents | 3 (1.3) |
| Antigout preparations | 3 (1.3) |
| Psychoanaleptics | 3 (1.3) |
| Other | 11 (<1) |
| Abbreviation: ATC = Anatomic Therapeutic Chemical classification | |

**TABLE**

**Table 1S.** Detailed participants concomitant therapies, according to second level Anatomic Therapeutic Chemical classification.
